# Double disadvantage: a case control study on health-related quality of life in children with sickle cell disease

**DOI:** 10.1186/1477-7525-8-121

**Published:** 2010-10-26

**Authors:** Channa T Hijmans, Karin Fijnvandraat, Jaap Oosterlaan, Harriët Heijboer, Marjolein Peters, Martha A Grootenhuis

**Affiliations:** 1 Psychosocial Department, Emma Children's Hospital, Academic Medical Center, P.O. Box 304, 1100 VC Amsterdam, The Netherlands; 2 Department of Pediatric Hematology, Emma Children's Hospital, Academic Medical Center, P.O. Box 304, 1100 VC Amsterdam, The Netherlands; 3 Department of Clinical Neuropsychology, VU University Amsterdam, Van der Boechorststraat 1, 1081 BT Amsterdam, The Netherlands

## Abstract

**Background:**

Low health-related quality of life (HRQoL) of children with sickle cell disease (SCD) may be associated with consequences of the disease, or with the low socio-economic status (SES) of this patient population. The aim of this study was to investigate the HRQoL of children with SCD, controlling for SES by comparing them to healthy siblings (matched for age and gender), and to a Dutch norm population.

**Methods:**

The HRQoL of 40 children with homozygous SCD and 36 healthy siblings was evaluated by the KIDSCREEN-52. This self-report questionnaire assesses ten domains of HRQoL. Differences between children with SCD and healthy siblings were analyzed using linear mixed models. One-sample t-tests were used to analyze differences with the Dutch norm population. Furthermore, the proportion of children with SCD with impaired HRQoL was evaluated.

**Results:**

In general, the HRQoL of children with SCD appeared comparable to the HRQoL of healthy siblings, while children with SCD had worse HRQoL than the Dutch norm population on five domains (Physical Well-being, Moods & Emotions, Autonomy, Parent Relation, and Financial Resources). Healthy siblings had worse HRQoL than the Dutch norm population on three domains (Moods & Emotions, Parent Relation, and Financial Resources). More than one in three children with SCD and healthy siblings had impaired HRQoL on several domains.

**Conclusion:**

These findings imply that reduced HRQoL in children with SCD is mainly related to the low SES of this patient population, with the exception of disease specific effects on the physical and autonomy domain. We conclude that children with SCD are especially vulnerable compared to other patient populations, and have special health care needs.

## Background

Sickle cell disease (SCD) is a hereditary red blood cell disorder that occurs predominantly in people of African ancestry [1]. SCD is becoming one of the most common genetic disorders in children in Western Europe, due to demographic changes [2]. In the Netherlands, an estimated number of 1000 children, originating from Surinam and Central Africa, have SCD. The disease is characterized by chronic haemolytic anaemia and vascular occlusion, causing recurrent painful episodes, irreversible organ damage, and neurocognitive deficits. Besides the medical consequences, most families with a child with SCD have to cope with financial and social problems, as the majority belongs to immigrant communities with a low socio-economic status (SES) and is single parented [3]. Nevertheless, the differential impact of low SES and the disease specific consequences of SCD on health-related quality of life (HRQoL) in children is not well known.

Quality of Life (QoL) is defined as an individual's perception of one's position in life in the context of culture and value systems, as well as in relation to one's goals, expectations, standards and concerns. HRQoL is defined as QoL in which a dimension of personal judgement of one's health and disease is added [4]. In the case of children, HRQoL is also influenced by factors such as the ability to participate in peer groups and the ability to keep up with developmental activities. Difficulties in measuring HRQoL in children include a lack of consensus on suitable (cross-cultural), self-report instruments and the need for different instruments in different age groups. Recently, the KIDSCREEN-52 was developed in Europe as a generic, cross-national HRQoL questionnaire that evaluates HRQoL regardless of whether children are in good health or suffer from a chronic medical condition [5]. This self-report questionnaire evaluates three components of children's well-being, in line with the definition of HRQoL of the World Health Organization: the physical, psychological, and social component [4].

Previous research has evaluated psychological and social problems in children with SCD. Although findings were inconclusive, internalizing problems (such as anxiety and depression), social problems, and feelings of low self-esteem are commonly reported [6-10]. However, the measurement of HRQoL, encompassing all three components of children's well-being, is still in its infancy in children with SCD [11]. For example, compared to over 400 studies on HRQoL in children with cancer, research on HRQoL of children with SCD is just scratching the surface [12]. The relatively few studies that have been performed revealed that HRQoL of children with SCD is generally poor [13-18].

However, these studies have several limitations. First, some investigators solely relied on parental ratings of HRQoL [14,15], while self report has been described as the ideal when measuring HRQoL [11]. Second, almost all previous studies have been performed in the United States of America. As the healthcare system and the origins of the patient population in the USA differ from Western Europe, findings from these studies cannot be generalized to the European population of children with SCD. Although the first study on HRQoL of children with SCD was performed at our own institution and showed lower HRQoL on several domains [13], this study did not yet consider the impact of low socio-economic status (SES) on HRQoL. Subsequent studies that did specifically address the role of low SES yielded conflicting results, which can be ascribed to isolated concepts of SES that were used (e.g. parental work status, educational level, neighbourhood socioeconomic distress, or family income) [14-17]. SES is defined as a total measure of a person's work experience and of an individual's or family's economic and social position relative to others, based on income, education, and occupation. A more comprehensive approach to grasp all these economic and sociological aspects of SES could be to compare children with SCD to a control group of healthy siblings with the same background. So far, no studies on HRQoL of children with SCD have included healthy siblings as controls. Instead, most researchers compared children with SCD to (non-White) population norms or to a control group with a different ethnicity or income level.

The aim of the current study was to examine whether reduced HRQoL in children with SCD is related to consequences of the disease or to the low SES of most patients. Therefore, we investigated self-reported HRQoL of children with SCD compared to (1) healthy siblings (who are comparable in age, gender, ethnicity and SES) and (2) a Dutch norm population. We hypothesized that children with SCD would have a lower HRQoL than healthy siblings with the same background, suggesting reduced HRQoL to be related to consequences of the disease. Alternatively, if children with SCD and healthy siblings would have comparable HRQoL, but children with SCD would have a lower HRQoL than the Dutch norm population, this would suggest that reduced HRQoL is related to low SES.

## Methods

### Participants

Forty children with SCD and 36 healthy siblings aged 6 to 18 years participated in the study. A total of 46 children were randomly selected from the children receiving treatment for a severe form of SCD (HbSS or HbS-β0-thalassemia) at the Comprehensive Sickle Cell Care Center of the Emma Children's Hospital, Academic Medical Center in Amsterdam. From these 46 children, 40 (87%) participated (6 declined). Most of them had an HbSS genotype (*n *= 36, 90%), the other four (10%) had an HbS-β0-genotype. The clinical condition of all children with SCD was stable at the time the HRQoL evaluation took place.

After recruitment of participants with SCD, healthy siblings of the group of participants were recruited. Fifty-seven healthy (full or half) siblings were invited to participate. From these, 36 (63%) participated. Siblings were matched for age and gender one by one to participants. As no sibling match was available for all participating children with SCD, we recruited nineteen healthy siblings (50%) in families from non-participating children with SCD receiving care at our hospital. These healthy siblings had similar demographic characteristics and were matched for age and gender one by one to participants as well. Inclusion took place between October 2007 and October 2008.

### Questionnaire

HRQoL was evaluated by the KIDSCREEN-52, a generic self-report questionnaire that uses questions derived from focus groups of children and adolescents across Europe. It is applicable for both healthy and chronically ill children and adolescents aged between 8 and 18 years. The KIDSCREEN-52 consists of 52 items assessing ten domains of HRQoL during the previous week: 'Physical Well-being', 'Psychological Well-being', 'Moods & Emotions', 'Self-Perception', 'Autonomy', 'Parent Relation & Home Life', 'Financial Resources', 'Social Support & Peers', 'School Environment' and 'Bullying'. Items are scored on a five-point scale. Within each domain, item scores are summed and transformed to a T value. Children in the European norm population have a mean score of 50 with a standard deviation (SD) of 10, with higher values indicating better HRQoL. This instrument is validated in a Dutch population of 1960 children, with an age distribution of 8 to 11 years (n = 641) and 12 to 18 years (n = 1270). Means of the Dutch norm population vary across domains, but are generally 2-3 points higher than in the European norm population. The instrument has satisfactory reliability and validity and good internal consistency [19].

### Procedure

The medical ethics committee of the Academic Medical Center of Amsterdam approved the study protocol. Written informed consent was obtained from parents and from children aged twelve years and older. Children were invited to visit our outpatient clinic where the KIDSCREEN-52 was administered. Questions were read aloud by an interviewer for children with lower reading capabilities. Young participants received cognitive debriefing to ascertain they understood the questions. All children were able to comprehend the instructions and reliably report their own HRQoL. Completion of the questionnaire required 20 minutes.

### Statistical analysis

The Statistical Package for the Social Sciences (SPSS version 16.0) was used to manage and analyze the data. First, missing values were handled according to the guidelines given in the manual of the KIDSCREEN-52. The percentage of missing data was <10%. Second, an independent t-test and Chi square tests were used to compare children with SCD to healthy siblings on demographic characteristics. As demographic characteristics of the Dutch norm population of the KIDSCREEN-52 were not available, data on marital status, educational level, and employment in the general Dutch population was obtained from the Central Dutch Bureau of Statistics CBS http://www.cbs.nl. Third, linear mixed models were used to analyze differences in HRQoL between children with SCD and healthy siblings, and one-sample t-tests were used to analyze differences in HRQoL compared to the Dutch norm population of the KIDSCREEN-52. The linear mixed model allows for the investigation of group differences while controlling for the non-independency of data (i.e. more than one child participated per family, which resulted in related measurements within groups and between groups). All domains were analyzed using group (patients or healthy siblings) as fixed factor and family as random effect to account for within family correlation.

Besides analyzing the total study sample, we performed exploratory analyses separately for children aged 6-11 and adolescents aged 12-18 years, as the implications of SCD for children's social, emotional and cognitive development may vary depending on the impact of the disease at each stage of development [20]. Although the KIDSCREEN-52 was originally designed for children 8-18 years, we included 4 children aged 6 and 8 children aged 7 years. We explored differences in HRQoL between children aged 6-7 (n = 12) and 8-18 years (n = 28) using independent t-tests, and performed all mixed model analyses with and without children aged 6-7 years. As no significant differences between these age groups were found, children aged 6-7 years were included in the final analyses.

Effect sizes (d) were calculated for differences between children with SCD and healthy siblings, by dividing the difference in mean score between children with SCD and healthy siblings by the pooled SD of both groups. According to Cohen [21], effect between 0.2 and 0.5 are considered small, effect sizes between 0.5 and 0.8 moderate, and effect sizes > 0.8 large. Furthermore, we calculated the point estimate of the mean difference and confidence intervals between scores of children with SCD and the Dutch norm population [22].

To add clinical meaning, we evaluated how many children had impaired HRQoL scores. We followed Varni et al who defined impaired HRQoL scores as ≥1 SD below the population mean [23]. The proportion of children with SCD with scores ≥1 SD below the Dutch population mean was compared to the proportion of healthy siblings with scores ≥1 SD below the Dutch population mean using Chi square test. Confidence intervals were calculated [22] for comparison of the proportion of children with SCD with impaired HRQoL to the proportion of children in the Dutch norm population. In the Dutch norm population, 16% of children have impaired HRQoL, based on the distribution of T values. A significance level of *p *< 0.05 was used for all tests. Considering the exploratory nature of our study, we did not correct for multiple comparisons.

## Results

### Demographics

Table 1 provides the demographic characteristics of children with SCD, healthy siblings, and the general Dutch population. Children with SCD did not differ significantly from healthy siblings in the distribution of age, gender, country of origin, parental marital status, maternal educational level or parental paid employment. However, compared to the general Dutch population, more children with SCD and healthy siblings grow up in single-parent families with lower educational levels and fewer paid employment.

**Table 1:** Demographic characteristics of children with SCD, healthy siblings and the general Dutch population

	Children with SCD (n = 40)		Healthy siblings (n = 36)		General population ^c^	
Age in years, M (*SD*) ^a^	11.7	(3.1)	11.6	(3.4)	-	-
Boys, *n *(%) ^a^	20	(50)	18	(50)	-	-
Country of origin ^a^						
Surinam, *n *(%)	18	(45)	23	(64)	-	-
West/Central Africa, *n *(%)	19	(48)	10	(28)	-	-
Turkey, *n *(%)	2	(5)	2	(5)	-	-
Netherlands Antilles, *n *(%)	1	(2)	1	(3)	-	-
Parental marital status ^a^					(n = 16.485) ^d^	
Married/living together, *n *(%)	15	(38)	17	(47)	2.051	(81)
Single, *n *(%)	25	(62)	19	(53)	474	(19)
Highest level of education of mother ^a b^					(n = 2.039) ^e^	
Lower, *n *(%)	22	(55)	19	(53)	431	(21)
Intermediate, *n *(%)	6	(15)	10	(28)	955	(47)
Higher, *n *(%)	2	(5)	1	(3)	641	(31)
Not specified, *n *(%)	10	(25)	6	(17)	13	(1)
Parental paid employment ^a^					(n = 3.928) ^f^	
Yes, *n *(%)	22	(55)	23	(64)	3.787	(96)
No, *n *(%)	16	(40)	9	(25)	141	(4)
Not specified, *n *(%)	2	(5)	4	(10)	-	-

^a ^Non-significant difference between children with SCD and healthy siblings.

^b ^Education: Lower: elementary education, general secondary education junior-level, lower vocational education; Intermediate: general secondary education-senior level, and vocational education-junior level; Higher: vocational education-senior level and university education.

^c ^Data obtained from the Central Dutch Bureau of Statistics CBS. All numbers × 10^3^.

^d ^Total Dutch population.

^e ^Total number of Dutch females aged 25-45.

^f ^Total number of Dutch people aged 25-45 who are part of the labour force.

### HRQoL of total study sample

Results are reported in Table 2. Compared to healthy siblings, children with SCD had significantly lower HRQoL on only 1 domain of the KIDSCREEN-52: Physical Well-being. The effect size for this domain was moderate. However, compared to the Dutch norm population, children with SCD scored significantly lower on 5 domains: Physical Well-being, Moods & Emotions, Autonomy, Parent Relation, and Financial Resources. Healthy siblings also scored significantly lower on Moods & Emotions, Parent Relation, and Financial Resources compared to the Dutch norm population.

**Table 2:** HRQoL of total study sample of children with SCD compared to healthy siblings and Dutch norm population of the KIDSCREEN-52

	Children with SCD (n = 40)		Healthy siblings (n = 36)		SCD versus healthy siblings	Norm population (n = 1960)		SCD versus norm population
	**Mean**	**SD**	**Mean**	**SD**	**Effect size**	**Mean**	**SD**	**Point estimate of mean difference (95% CI)**

Physical Well-being	49 †*	8.7	54	11.4	0.5	53	10.0	-4 (-7; -1)
Psychological Well-being	53	9.3	55	9.6	0.2	53	8.8	0 (-3; +3)
Moods & Emotions	47 *	8.9	47 *	10.2	0	51	9.6	-4 (-7; -1)
Self-Perception	52	8.8	53	10.8	0.1	52	10.1	0 (-3; +3)
Autonomy	48 *	8.1	52	9.4	0.5	55	9.1	-7 (-10; -4)
Parent Relation	50 *	10.3	50 *	10.4	0	53	9.1	-3 (-6; 0)
Financial Resources	47 *	10.0	46 *	11.8	0.1	52	9.7	-5 (-8; -2)
Social Support & Peers	50	11.3	51	12.4	0.1	52	9.3	-2 (-5; 0)
School Environment	54	8.6	54	10.0	0	53	10.0	1 (-2; +4)
Bullying	45	11.5	47	11.5	0.6	49	10.4	-4 (-7; -1)

Higher scores represent better HRQoL.

† p < 0.05 children with SCD versus healthy siblings.

*p < 0.05 children with SCD and/or healthy siblings versus Dutch norm population of the KIDSCREEN-52.

### HRQoL of children aged 6-11

Results are reported in Table 3. Although children with SCD aged 6-11 years had slightly lower mean scores than healthy siblings on 8 of the 10 domains of the KIDSCREEN-52, no statistically significant differences were found. There was a moderate effect size for lower HRQoL of children with SCD on Physical Well-being, compared to healthy siblings. Effect sizes for the other domains were small.

**Table 3:** HRQoL of children with SCD aged 6-11 compared to age matched healthy siblings and Dutch norm population of the KIDSCREEN-52

	Children with SCD (n = 17)		Healthy siblings (n = 19)		SCD versus healthy siblings	Norm population (n = 641)		SCD versus norm population
	**Mean**	**SD**	**Mean**	**SD**	**Effect size**	**Mean**	**SD**	**Point estimate of mean difference (95% CI)**

Physical Well-being	51 *	9.5	58	11.7	0.7	57	9.5	-6 (-11; +1)
Psychological Well-being	55	8.7	57	9.1	0.2	56	9.1	-1 (-5; +3)
Moods & Emotions	46 *	11.0	48	12.3	0.2	53	9.5	-7 (-12; -2)
Self-Perception	50 *	7.9	54	11.0	0.4	57	9.8	-7 (-12; -2)
Autonomy	49 *	6.8	50 *	9.4	0.1	57	8.6	-8 (-12; -4)
Parent Relation	48 *	10.4	50 *	10.1	0.2	56	8.4	-8 (-12; -4)
Financial Resources	44 *	11.8	43 *	14.2	0.1	51	10.6	-7 (-12; -2)
Social Support & Peers	53	11.7	49	13.9	0.3	53	9.1	0 (-4; +4)
School Environment	56	8.1	57	11.2	0.1	58	10.2	-2 (-7; +3)
Bullying	42 *	10.9	46	12.6	0.3	48	11.0	-6 (-11; +1)

Higher scores represent better HRQoL.

*p < 0.05 children with SCD and/or healthy siblings versus Dutch norm population of the KIDSCREEN-52.

Compared to the Dutch norm population, children with SCD aged 6-11 years had significantly lower HRQoL on 7 of the 10 domains: Physical Well-being, Moods & Emotions, Self-Perception, Autonomy, Parent Relation, Financial Resources, and Bullying. Healthy siblings had significantly lower mean scores on 3 of these domains: Autonomy, Parent Relation, and Financial Resources.

### HRQoL of children aged 12-18

Results are reported in Table 4. Adolescents with SCD had a significantly lower HRQoL on Autonomy compared to healthy siblings. The effect size for this domain was moderate. Adolescents with SCD also had significantly lower scores on Autonomy compared to the Dutch norm population. No other significant differences compared to the Dutch norm population were found. Healthy siblings aged 12-18 years did no differ significantly from the Dutch norm population either, except for significantly lower scores on Moods & Emotions.

**Table 4:** HRQoL of adolescents with SCD aged 12-18 compared to age matched healthy siblings and Dutch norm population of the KIDSCREEN-52

	Children with SCD (n = 23)		Healthy siblings (n = 17)		SCD versus healthy siblings	Norm population (n = 1270)		SCD versus norm population
	**Mean**	**SD**	**Mean**	**SD**	**Effect size**	**Mean**	**SD**	**Point estimate of mean difference (95% CI)**

Physical Well-being	48	8.0	50	9.5	0.2	50	9.4	-2 (-6; +2)
Psychological Well-being	51	9.5	53	9.7	0.2	52	8.4	-1 (-5; +3)
Moods & Emotions	48	7.2	46 *	7.4	0.3	51	9.6	-3 (-7; +1)
Self-Perception	54	9.2	52	10.9	0.2	50	9.3	4 (0; +8)
Autonomy	47 † *	9.0	53	9.5	0.7	54	9.1	-7 (-11; -3)
Parent Relation	51	10.2	49	11.0	0.2	52	9.3	-1 (-5; +3)
Financial Resources	50	7.8	50	7.0	0	53	9.2	-3 (-7; +1)
Social Support & Peers	48	10.8	53	10.4	0.5	52	9.4	-4 (-8; 0)
School Environment	53	8.8	49	6.4	0.5	51	8.7	2 (-2; +6)
Bullying	48	11.6	49	10.5	0.1	49	10.1	-1 (-5; +3)

Higher scores represent better HRQoL.

† p < 0.05 children with SCD versus healthy siblings.

*p < 0.05 children with SCD and/or healthy siblings versus Dutch norm population of the KIDSCREEN-52.

### Proportion of children with impaired HRQoL

**Figure 1:**
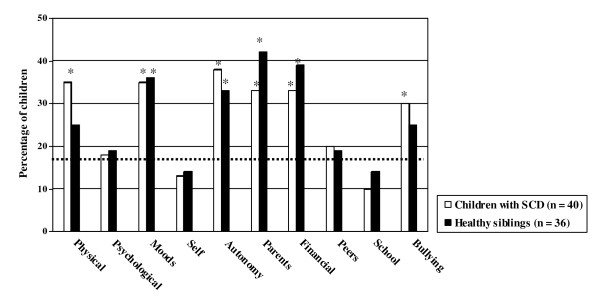
**Proportions of children with SCD and healthy siblings with impaired HRQoL (≥1 SD below the mean of the Dutch norm population of the KIDSCREEN-52)**. The dotted line represents the 16% of children in the Dutch norm population of the KIDSCREEN-52 with impaired HRQoL. * 95% Confidence interval of the proportion of children with SCD or healthy siblings with impaired HRQoL exceeds the proportion in the Dutch norm population.

Results are reported in Figure 1. The proportion of children with SCD with impaired HRQoL (≥1 SD below the mean) was similar to the proportion of healthy siblings with impaired HRQoL. However, the proportion of children with SCD with impaired HRQoL was significantly larger than the proportion in the Dutch norm population on Physical Well-being, Moods & Emotions, Autonomy, Parent Relation, Financial Resources, and Bullying. More than 1 in 3 children with SCD (between 30 - 38%) had impaired HRQoL on these domains, which is a twofold increase in comparison to the Dutch norm population (16%). The proportion of healthy siblings with impaired HRQoL was also significantly larger than the proportion in the Dutch norm population on Moods & Emotions, Autonomy, Parent Relation, and Financial Resources.

## Discussion

This is the first study to assess multidimensional HRQoL in children with SCD using a self-report measure and a control group of healthy siblings. In general, the HRQoL of children with SCD appeared similar to the HRQoL of healthy siblings, with the exception of lower scores on the physical domain for children with SCD, and, specifically in adolescents, lower scores on the autonomy domain. However, the HRQoL of both children with SCD and healthy siblings was considerably lower than the Dutch norm population on several domains, specifically in children aged 6-11. This implies that reduced HRQoL in children with SCD is primarily related to the low SES of this patient population, although there are disease specific effects on the physical and autonomy domain.

A large study in urban elementary schoolchildren from poor socioeconomic areas formerly established the essential impact that SES can have on HRQoL. These children reported lower HRQoL than chronically diseased children [24]. Nevertheless, prior studies in children with SCD suggested that the HRQoL of children with SCD is impaired, even after considering the potential detrimental effect of low SES [15-17]. The contrast between these previous findings and the present results may be explained by the use of different instruments, different comparison groups and particularly the use of proxy- versus self-report. In our study, a self-report questionnaire was administered, while other researchers have mainly used parental proxy-report. Parents of chronically diseased children generally tend to report worse HRQoL for their children than children themselves [25,26], which was confirmed by previous studies in children with SCD [13,16,17]. This may have led to an underestimation of the HRQoL of children with SCD. Parents could underestimate their children's HRQoL as a result of parental distress [27]. Alternatively, parents and children could have different perceptions: while children may be unaware of the potential consequences of the disease, parents may have greater concerns for their future [28] and take on a protective attitude.

Parental (over-)protection could also underlie our finding of lower scores of adolescents with SCD on the autonomy domain, compared to healthy siblings. This finding is congruent with results of a previous study in our hospital [13] as well as other studies on adjustment in adolescents with SCD [29,30], and seems to be associated with consequences of the disease. Therefore, we recommend that parental overprotection should be considered and targeted in intervention programs for adolescents with SCD. As higher levels of self-efficacy were previously found to be associated with fewer SCD symptoms [30], interventions to increase autonomy could possibly lead to a decrease in SCD symptomatology in this age group.

Interestingly, adolescents did not differ significantly from healthy siblings or the Dutch norm population on any of the other domains except for autonomy, while children aged 6-11 did have significantly lower scores than the norm population on 7 of the 10 domains. Although adolescents have been described to generally experience more problems in psychosocial adaptation than younger children [7], HRQoL scores of adolescents were within the normative range in a previous study as well [27]. The resilience of adolescents could be a consequence of developmental growth and adjustment, possibly causing a better coping style with increasing age. This should be investigated further longitudinally.

Surprisingly, children with SCD did not report any social problems. This is a remarkable contrast to the results of previous findings [6-10], including results of our own work in which parents rated their children with SCD to show less competent social behavior than healthy siblings [31]. Furthermore, it is striking that children with SCD did not report a lower HRQoL on the School environment domain, as studies on neurocognitive sequelae of SCD have shown a decrease in general intellectual ability as well as deficits in specific neurocognitive domains in children with SCD [32]. Children with SCD may have adjusted to their social and cognitive deficits and therefore do not subjectively experience HRQoL problems in these domains. In future research behavioural and neurocognitive data should be correlated to HRQoL, to gain more insight into the relation between objectively identified problems and the subjective HRQoL of children with SCD.

While interpreting the results of this study, strengths and limitations should be taken into account. Findings of this study are strengthened by the use of a well-standardized self-report measure and robust statistical methods to take within family correlations into account. Furthermore, by including a control group of healthy siblings with the same age, ethnicity and SES, the differential effect of low SES and disease specific consequences of SCD on HRQoL could be determined.

However, not all participating children with SCD had an eligible sibling, causing us to recruit healthy siblings from the entire SCD patient population. This led to an overrepresentation of children from Surinam descent in the healthy sibling group. Nevertheless, demographic differences between the groups were not statistically significant and explorative within-group analyses revealed no significant differences between children from Surinam or African descent on any of the HRQoL domains. Another limitation was the small sample size, mitigating statistical power. The results of our exploratory analyses need to be interpreted within this limitation. Moreover, the current study design did not allow us to investigate if there is an interaction effect of SES and sickle cell disease on HRQoL. Further limitations concerned the instrument we used to assess HRQoL. The KIDSCREEN-52 is not yet validated in children aged 6 and 7. However, young participants received cognitive debriefing to ascertain reliable self-report, and this issue was taken into account in statistical analyses. Children as young as 5 years of age have been found to be able to reliably report HRQoL [26,33,34]. Furthermore, data were collected in a clinical setting, which may have affected the response of the participants. Finally, the KIDSCREEN-52 assesses HRQoL of the past week. As SCD has a very unpredictable course, it would be more appropriate to assess HRQoL over longer time periods, e.g. the past month. These issues should be taken into account in future research.

## Conclusion

Based on the present findings, we conclude that children with SCD are primarily disadvantaged by their low SES, causing lower HRQoL on several domains compared to the Dutch norm population. Nevertheless, they are also affected by specific consequences of the disease, reflected by the lower HRQoL on the physical and autonomy domains compared to healthy siblings. Moreover, one in three children with SCD experience impaired HRQoL on several domains. As children with SCD are not only affected by their disease but also by their low SES, children with SCD seem to be especially vulnerable compared to other patient populations, and need specific care in the hospital. Therefore, we argue for routine monitoring of HRQoL in children with SCD. Incorporating patient reported outcomes of HRQoL in daily clinical practice will contribute to better communication with health care professionals. This can provide these children with the additional care they need due to their double disadvantage: a chronic disease, on top of an unfavourable background.

## Declaration of competing interests

The authors declare that they have no competing interests.

## Authors' contributions

CTH collected the data, analyzed and interpreted the data and drafted the manuscript. KF designed and supervised execution of the study, interpreted the data and drafted and revised the manuscript. JO, HH and MP supervised execution of the study and revised the manuscript. MAG designed and supervised execution of the study, interpreted the data and drafted and revised the manuscript. All authors read and approved the manuscript.
